# Understanding Biological Aging in Latin America: A Systematic Review of External Exposures and Their Impact on Telomere Length and Epigenetic Clocks

**DOI:** 10.1002/alz70860_103053

**Published:** 2025-12-23

**Authors:** Ellen Riquelme Sepúlveda, Liset Gonzalez, Claudia Duran‐Aniotz, Agustin Ibanez, Carolina Ochoa‐Rosales

**Affiliations:** ^1^ Center for Social and Cognitive Neuroscience (CSCN), Santiago, Región Metropolitana, Chile; ^2^ Latin American Brain Health Institute (BrainLat), Santiago, Región Metropolitana, Chile; ^3^ Latin American Brain Health Institute (BrainLat), Universidad Adolfo Ibañez, Santiago, Chile; ^4^ Latin American Institute for Brain Health (BrainLat), Universidad Adolfo Ibañez, Santiago, Metropolitan Region, Chile; ^5^ Latin American Brain Health Institute (BrainLat), Universidad Adolfo Ibañez, Santiago, Region Metropolitana, Chile

## Abstract

**Background:**

Aging is a risk factor for several human diseases. Genetic and epigenetic biomarkers such as telomere length (TL) and DNA methylation‐based epigenetic clocks help assess biological aging and disease risk. The exposome— covering lower socioeconomic factors, unhealthy lifestyles, pollution, and adverse early experiences —promotes accelerated biological aging, but research on these effects in Latin America, a region exposed to a higher burden of health risk factors, remains limited. We systematically reviewed the evidence on the relationship of various exposures with TL or epigenetic clocks biomarkers in this region.

**Method:**

We searched in PubMed, Scopus and Scielo, following the PRISMA guidelines. Keywords included social determinants of health, socioeconomic status, deprivation, education, educational attainment, income, pollution, violence, discrimination, early life experience, childhood experiences, adverse experience, lifestyle, behavior, diet, nutrition, physical activity, exercise, sleeping, smoking, alcohol, alcohol consumption, and each country in Latin America, covering telomere length and epigenetic clocks.

**Result:**

Fourteen observational studies were selected, from Brazil, Costa Rica, Chile, Honduras, Nicaragua, Bolivia, Panama and Mexico, with samples ranging from 83 to 507 participants. One study assessed epigenetic clocks, showing that a combination of adequate sleep and late bedtimes was related to accelerated aging, while short sleep combined with high sedentary behavior in females showed an age deceleration. For TL, food insecurity in fathers, lower education in men, and childhood food deprivation in women were linked to shorter TL, similar to smoking. Diets rich in fruits, vegetables, and beans, and experiencing childhood poverty and violence correlated with longer TL. Arsenic exposure in indigenous women was linked to longer TL, PM2.5 exposure in pregnancy correlated with shorter TL. Maternal lead exposure during pregnancy had no impact on TL in the offspring, similar to alcohol abuse and adverse intrauterine conditions.

**Conclusion:**

This is the first study to summarize the evidence on the relationship of the exposome and LT and epigenetic clocks in the Latin American population. Exposures like socioeconomic adversity, diet, pollution, and lifestyle behaviors may impact biological aging.

